# Apigenin in cancer therapy: anti-cancer effects and mechanisms of action

**DOI:** 10.1186/s13578-017-0179-x

**Published:** 2017-10-05

**Authors:** Xiaohui Yan, Miao Qi, Pengfei Li, Yihong Zhan, Huanjie Shao

**Affiliations:** Key Laboratory of the Ministry of Education for Medicinal Plant Resources and Natural Pharmaceutical Chemistry, College of Life Science, Shaanxi Normal University, Xi’an, 710119 China

**Keywords:** Apigenin, Anti-cancer, Mechanism of action, Combination therapy

## Abstract

Apigenin is a common dietary flavonoid that is abundantly present in many fruits, vegetables and Chinese medicinal herbs and serves multiple physiological functions, such as strong anti-inflammatory, antioxidant, antibacterial and antiviral activities and blood pressure reduction. Therefore, apigenin has been used as a traditional medicine for centuries. Recently, apigenin has been widely investigated for its anti-cancer activities and low toxicity. Apigenin was reported to suppress various human cancers in vitro and in vivo by multiple biological effects, such as triggering cell apoptosis and autophagy, inducing cell cycle arrest, suppressing cell migration and invasion, and stimulating an immune response. In this review, we focus on the most recent advances in the anti-cancer effects of apigenin and their underlying mechanisms, and we summarize the signaling pathways modulated by apigenin, including the PI3K/AKT, MAPK/ERK, JAK/STAT, NF-κB and Wnt/β-catenin pathways. We also discuss combinatorial strategies to enhance the anti-cancer effect of apigenin on various cancers and its use as an adjuvant chemotherapeutic agent to overcome cancer drug resistance or to alleviate other adverse effects of chemotherapy. The functions of apigenin against cancer stem cells are also summarized and discussed. These data demonstrate that apigenin is a promising reagent for cancer therapy. Apigenin appears to have the potential to be developed either as a dietary supplement or as an adjuvant chemotherapeutic agent for cancer therapy.

## Background

Cancer is a disease caused by the abnormal proliferation and differentiation of cells and is governed by tumorigenic factors. Cancer is the second most common cause of human death worldwide. Currently, chemotherapy is still one of the best therapeutic methods to treat cancer. With wider application and further understanding, the side effects and acquired drug resistance of synthesized small molecule compounds have caused more and more concerns [1, 2]. Therefore, natural and edible small molecules such as flavones, which are thought to have remarkable physiological effects, low toxicity and non-mutagenic properties in the human body, have gained more and more interest in anti-cancer agent development.

Apigenin, known chemically as 4′,5,7-trihydroxyflavone, belongs to the flavone subclass and is abundant in vegetables, fruits and beverages, such as parsley, grapes, apples, chamomile tea and red wine. Apigenin is also one of the active ingredients in Chinese medicinal herbs. In its natural form, apigenin is usually conjugated to a glycoside in vivo. Apigenin was classified as a class II drug of Biopharmaceutical Classification System in a recent study [3]. It has a poor solubility in aqueous phase but high intestinal permeability determined by single-pass intestinal perfusion technique. For in vivo studies, oral administration of apigenin at 60 mg/kg in rat resulted in low blood levels, with a C_max_ of 1.33 ± 0.24 μg/mL and AUC 0–t° of 11.76 ± 1.52 μg h/mL. With novel carbon nanopowder drug carrier system of solid dispersions, the relative oral bioavailability of apigenin was enhanced by approximately 183% [4]. To better understand the pharmacokinetics and distribution of apigenin in vivo, Wistar rats were treated once with radiolabeled apigenin by oral administration. Then the potential storage tissue and blood kinetic were analyzed. Results showed that 51.0% of radioactivity was recovered in urine, 12.0% in feces, 1.2% in the blood, 0.4% in the kidneys, 9.4% in the intestine, 1.2% in the liver, and 24.8% in the rest of the body within 10 days. Furthermore, blood kinetics analysis indicated that radioactivity appeared at 9 h and reached a maximum at 24 h post-ingestion time, suggesting a slow distribution phase and slow elimination. Thus a possible accumulation of apigenin in the body is hypothesized [5].

**Fig. 1:**
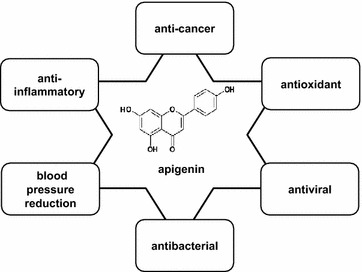
Molecular structure and physiological functions of apigenin

Apigenin has been used as a traditional medicine for centuries because of its physiological functions as an antioxidant and anti-inflammatory [6, 7], its role in lowering blood pressure [8], and its antibacterial and antiviral properties [9]. In addition to those effects, apigenin was proven to have tumor suppression efficacy in the last few decades (Fig. 1). Since Birt et al. first reported that apigenin had anti-cancer activities in 1986 [10], more and more evidence has been presented to demonstrate that apigenin shows antitumor efficacy against various types of cancer with both cell lines in vitro and mouse models in vivo.

Apigenin has been demonstrated to show broad anti-cancer effects in various types of cancers, including colorectal cancer, breast cancer, liver cancer, lung cancer, melanoma, prostate cancer and osteosarcoma [11–16]. This flavone inhibits cancer cell proliferation by triggering cell apoptosis, inducing autophagy and modulating the cell cycle. Apigenin also decreases cancer cell motility and inhibits cancer cell migration and invasion. Recently, apigenin was reported to show anti-cancer activities by stimulating an immune response [17]. During those processes, multiple signaling pathways and protein kinases are modulated by apigenin, including PI3K/AKT, MAPK/ERK, JAK/STAT, NF-κB and Wnt/β-catenin.

In this review, we focus on apigenin’s antitumor effects and summarize the advancements in the anti-cancer effects of apigenin and its multiple underlying mechanisms that have been identified in recent years. We also discuss combinatorial strategies to enhance the anti-cancer effect of apigenin on various cancers using in vitro and in vivo models. Our purpose is to highlight apigenin as a promising agent for cancer therapy.

## Apigenin in cancer therapy

**Fig. 2:**
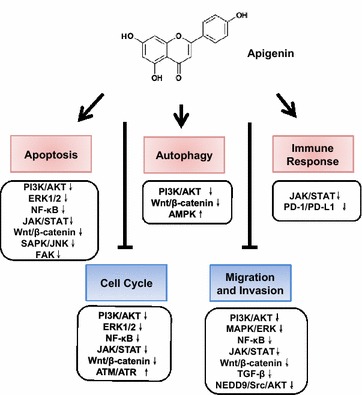
Apigenin triggers cell apoptosis, autophagy and immune response and inhibits cell cycle progress and cell migration and invasion by targeting multiple signaling pathways. Bold arrows of ↙ represents induction and ⊥ represents suppression of effects. Light arrow ↑ represents upregulation and ↓ represents downregulation of molecules pathways

Carcinogenesis is a multistage process and involves a series of genetic and epigenetic changes that lead to the initiation, promotion and progression of cancer. The strategies to treat cancer are to eliminate tumor cells by triggering cell apoptosis or to inhibit cancer cell proliferation by inducing cell cycle arrest, thereby making cancer a chronic disease and prolonging the survival of patients. Current strategies include the induction of apoptosis or autophagy, regulation of the cell cycle, inhibition of tumor cell migration and invasion, and stimulation of the immune response of patients. Thus far, apigenin has demonstrated all these antitumor activities with different tumor types in vitro and in vivo. Those anti-cancer effects of apigenin and the underlying signaling pathways involved are summarized, as in Fig. 2 and Table 1.

**Table 1:** Effects of apigenin treatment alone on cancer cells

Tumor type	Cell lines (concentration)	Mice (dosages)	Therapeutic effects	Mechanisms	Citations
Colorectal cancer	SW480 (40 μM)		Inhibited proliferation, invasion and migration	Inhibited Wnt/β-catenin signaling	[11]
	HCT116 (25 μM)		Inhibited proliferation; autophagy; apoptosis	Suppressed the expression of cyclin B1, Cdc2 and Cdc25c; induced PARP cleavage; induced LC3-II	[28]
	DLD1 and SW480 (40 μM)	20 mg/kg (athymic nude mice, intraperitoneally)	Inhibited proliferation, invasion and migration	Attenuated NEDD9; reduced phosphorylations of FAK, Src, and Akt	[48]
	SW480, DLD-1, and LS174T (40 μM)	50 mg/kg (BALB/c-nude mice, orthotopically implanted)	Inhibited proliferation, invasion and migration	Up-regulated TAGLN; down-regulated MMP-9 expression; decreasing phosphorylation of Akt	[50]
Breast cancer	BT-474 (40 μM)		Inhibited cell proliferation; apoptosis	Reduced the p-JAK1, p-JAK2 and p-STAT3; up-regulated the levels of cleaved caspase-8, cleaved caspase-3 and the cleavage of PARP	[25]
	MDA-MD-231 (40 μM)	5, 25 mg/kg (BALB/c-nude mice, orthotopically injected)	Cell cycle arrest	Suppressed cyclin A, cyclin B, and CDK1; upregulated p21^WAF1/CIP1^; inhibited HDAC activity; induced histone H3 acetylation	[29]
	MDA-MB-231 and T47D (40 μM)		Inhibited cell proliferation; apoptosis	Increased levels of caspase3, PARP cleavage and Bax/Bcl-2 ratios	[41]
	MDA-MB-468 and 4T1 (30 μM)		Enhanced the immune responses	Inhibited IFN-γ-induced PD-L1 expression; inhibited STAT1	[58]
	SKBR3 (40 μM)		Apoptosis	Reduced the expression of p-JAK2 and p-STAT3; inhibited VEGF	[98]
	MDA-MB-453 (60 μM)		Inhibited cell proliferation; apoptosis	Up-regulated caspase-8, caspase-3 and the cleavage of PARP; inactivation of JAK2 and STAT3	[99]
Lung cancer	H1299 and H460 (20 μM)		Inhibited cell proliferation; apoptosis	Suppressed GLUT1	[13]
	A549 (40 μM)		Inhibited cell proliferation, migration, invasion	Decreased the PI3K/Akt signaling pathway	[47]
Prostate cancer	LNCaP (20 μM)		Inhibited cell proliferation; apoptosis	Decreased cyclin D1, D2 and E; upregulated WAF1/p21	[15]
	PC-3 and DU145 (20 μM)	20, 50 μg/mouse/day (athymic nude mice, oral gavage)	Cell cycle arret; apoptosis	Suppression of XIAP, c-IAP1, c-IAP2 and survivin; decreased Bcl-xL and Bcl-2 and increase in Bax protein	[22]
	DU145 (20 μM)		Inhibited migration and invasion; cell cycle arrest	Increased E-cadherin; decreased snail and vimentin	[46]
		20 and 50 μg/mouse/day (TRAMP mice, oral gavage)	Inhibited tumorigenesis	Inhibited IKK activation and restored the expression of IκBα	[89]
	PC-3 and 22Rv1 (20 μM)	20 and 50 μg/mouse/day (athymic nude mice, oral gavage)	Inhibited cell proliferation, invasivion	Inactivation of IKKα; suppressed NF-ĸB/p65 activation	[90]
	PC3-M and LNCaP C4-2B (25 μM)		Inhibited cell proliferation and metastases	Inhibited the Smad2/3 and Src/FAK/ Akt pathways	[110]
	PC3 (25 μM)		Apoptosis; cell cycle arrest; suppressed stem cell migration	Increased p21 and p27; upregulated caspases-8, -3 and TNF-α; downregulation of PI3K/Akt and NF-κB signaling	[65]
Melanoma	A375, C8161 (40 μM)		Inhibited proliferation and invasion; apoptosis; cell cycle arrest	Activation of cleaved caspase-3 and cleaved PARP; decreased ERK1/2 proteins, p-AKT and p-mTOR	[14]
	A2058, A375 (20 μM)		Inhibited metastasis	Inhibited the phosphorylation of FAK/ERK1/2	[82]
	A375, G361 (20 μM)	150 mg/kg (C57BL/6 mice, oral gavage)	Inhibited metastasis	Suppressed STAT3 phosphorylation; down-regulated MMP-2, MMP-9, VEGF and Twist1	[97]
Leukemia	HL60 (60 μM)		Apoptosis	Activation of caspase-9 and caspase-3	[23]
	HL60 (50 μM); TF1 (30 μM)		Cell cycle arrest	Inhibited JAK/STAT pathway	[37]
	U937 (40 μM)	20, 40 mg/kg (athymic nude mice, intraperitoneally)	Apoptosis	Inactivation of Akt; activation of JNK; downregulated Mcl-1 and Bcl-2	[112]
Ovarian cancer	A2780 (20, 40 μM)	5 mg/kg (BALB/c nude mice, intraperitoneally)	Inhibited adhesion, migration and invasion	Inhibited FAK expression	[49]
	SKOV3 (20, 40 μM)		Inhibited the self-renewal capacity	Downregulated Gli1; inhibition of CK2α	[66]
Glioblastoma	GL-15 (50 μM)		Inhibited angiogenic	Reduced TGF-b1 production	[111]
	U87MG and U373MG (25 μM)		Inhibited self-renewal capacity	Blocked the activation of c-Met signaling	[64]
Renal cell carcinoma	ACHN, 786-0, and Caki-1 (20 μM)	30 mg/kg (BALB/c-nude mice intraperitoneally)	Cell cycle arrest	p53 accumulation; modulated ATM signalling	[30]
Adenoid cystic carcinoma	ACC-2 (40 μM)		Inhibited proliferation; apoptosis	Suppressed the expression of GLUT-1	[31]
Papillary thyroid carcinoma	BCPAP (25 μM)		Cell cycle arrest; autophagy	Down-regulation of Cdc25C expression	[32]
Oral squamous cell carcinoma	SCC-25, HaCaT (100 μM)		Inhibited proliferation; apoptosis	Decreased expression of cyclin D-1 and E; inactivation of CDK1	[33]
Pancreatic cancer	Murine Panc02 (20 μM)	25 mg/kg (female C57BL/6N mice, intraperitoneally)	Maintain T cell homeostasis	Stabilizing Ikaros expression	[60]
Mesothelioma	Malignant mesothelioma (MM) cells (50 μM)	20 mg/kg (C57BL/6 mice, oral gavage)	Apoptosis	Inhibited AKT and c-Jun phosphorylation, and inhibited NF-κB nuclear translocation	[91]
Osteosarcoma	U2OS and MG63 (50 μg/ml)		Inhibited proliferation and invasion	Inactivated Wnt/β-catenin signaling	[108]
Head and neck squamous cell carcinoma	HSC-3, HN-8, and HN- 30 (40 μM)		Suppressed cancer stem cell marker expression	Downregulated the stem cell markers of CD44,NANOG, and CD105, and abolished the hypoxia-induced increase	[63]
Cervical cancer	HeLa (40 μM)		Inhibited cell self-renewal capacity	Downregulation of CK2α expression	[67]

### Induction of apoptosis

Apoptosis is the process of programmed cell death. Apoptosis involves energy-dependent cascade events and different distinct morphological characteristics [18]. To date, apoptosis is induced by two core pathways: the extrinsic (death receptor) pathway and the intrinsic (mitochondrial) pathway. Apoptosis is a critical process that allows undesirable cells to be removed under physiological conditions. Avoiding apoptosis is one of the most important characteristics of cancer cells that makes them different from normal cells. Thus, triggering cancer cell apoptosis by targeting apoptotic pathways with chemotherapy reagents is a widely used strategy to treat cancer. Apigenin has been demonstrated to be an effective agent for triggering apoptosis via either the intrinsic or extrinsic pathway in human cancer cells.

The intrinsic apoptotic pathway is regulated by the Bcl-2 family of proteins, such as Bcl-2, Bcl-xL, Bcl-w and Mcl-1, which block apoptosis, while Bad, Bak, Bax, Bid and Bim trigger apoptosis [19–21]. Apigenin functions to upregulate pro-apoptotic proteins and/or downregulate pro-survival members, thereby inducing the intrinsic apoptotic pathway. In prostate cancer therapy, treatment of the androgen-refractory human prostate cancer cell lines PC-3 and DU145 with apigenin resulted in apoptosis and a reduction in cell viability caused by a decrease in Bcl-2 and Bcl-xL and an increase in the active form of the Bax protein, accompanied by dose-dependent suppression of XIAP, c-IAP1, c-IAP2 and survivin proteins [22]. In addition, in human promyelocytic leukemia HL-60 cells, apigenin reduced the mitochondrial outer membrane potential, released cytochrome c from the mitochondria into the cytosol, induced procaspase-9 processing, and finally induced cell apoptosis through the intrinsic apoptotic pathway [23]. In other reports, apigenin caused apoptosis by changing the ratio of pro-apoptotic to pro-survival mitochondrial proteins. Apigenin increased the Bax/Bcl-2 ratio in favor of cell apoptosis in prostate cancer cells [15]. Clearly, apigenin alone is able to trigger mitochondria-dependent apoptosis in various types of cancer cells.

Moreover, apigenin can enhance chemotherapy-induced cell apoptosis by modulating the expression level of mitochondrial proteins. In the colorectal cancer cell lines HCT116 and DLD1, apigenin upregulated Bim expression and downregulated Mcl-1 expression, thereby synergizing with the Bcl-2 inhibitor ABT-263 to trigger mitochondria-dependent cell apoptosis [24].

In addition to cases where apigenin triggered the intrinsic apoptotic pathway, apigenin was found to induce cell apoptosis via the extrinsic pathway or both the extrinsic and intrinsic pathways. Seo et al. found that apigenin neither affected the levels of Bcl-2 and Bax nor decreased the mitochondrial membrane potential in the human breast cancer BT-474 cells, but this compound induced extrinsic, caspase-dependent apoptosis by upregulating the levels of cleaved caspase-8 and cleaved caspase-3 [25]. In non-small cell lung cancer (NSCLC) cells, Chen et al. showed that apigenin upregulated the levels of death receptor 4 (DR4) and death receptor 5 (DR5) in a p53-dependent manner, thereby sensitizing NSCLC cells to TRAIL-induced apoptosis. Meanwhile, apigenin triggered the intrinsic apoptotic pathway by upregulating the pro-apoptotic proteins Bad and Bax and downregulating the anti-apoptotic proteins Bcl-xL and Bcl-2 [26]. Moreover, in human keratinocytes and organotypic keratinocytes, apigenin increased UVB-induced apoptosis via both the intrinsic and extrinsic apoptotic pathways as well. Apigenin caused changes in Bax localization and in the release of cytochrome c. Overexpression of the pro-survival protein Bcl-2 and the dominant-negative form of Fas-associated death domain protected against apigenin-induced apoptosis [27].

### Modulation of the cell cycle

Uncontrolled and rapid cell division is another hallmark of cancer. A number of natural compounds that induce cell cycle arrest have been proved effective for suppressing cancers in vitro, in vivo and in clinical settings. As evidenced, apigenin inhibits cancer cell proliferation by modulating the cell cycle and blocking the cell phase at the G2/M or G0/G1 checkpoint.

In human colorectal carcinoma HCT116 cells, apigenin treatment potently inhibited cell growth by inducing cell arrest at G2/M phase, associated with suppression of both cyclin B1 and its activating partners, Cdc2 and Cdc25c, and increase of cell cycle inhibitors, p53 and p21^WAF1/CIP1^ [28]. As in the human breast cancer cell line MDA-MB-231, Western blotting showed that the expression of cyclin A, cyclin B, and cyclin-dependent kinase-1 (CDK1) was suppressed by apigenin treatment. In addition, apigenin upregulated p21^WAF1/CIP1^ and increased the interaction of p21^WAF1/CIP1^ with proliferating cell nuclear antigen (PCNA), which inhibits cell cycle progression at the G2/M stage [29]. In addition, in renal cell carcinoma cells, apigenin caused DNA damage in ACHN cells in a time- and dose-dependent manner and induced G2/M phase cell cycle arrest through ataxia telangiectasia mutated (ATM) signal modulation [30]. In adenoid cystic carcinoma (ACC), apigenin induced G2/M-phase arrest and inhibited ACC-2 cell growth and proliferation in a dose- and time-dependent manner by decreasing the expression of Glucose transporter-1 (GLUT-1) [31]. And in human papillary thyroid carcinoma BCPAP cells, apigenin treatment caused G2/M cell cycle arrest via down-regulation of Cdc25c expression and stimulated the accumulation of reactive oxygen species (ROS) production, leading to induction of DNA damage [32].

Moreover, apigenin can induce cell cycle arrest at the G0/G1 or S checkpoints as well. In human prostate cancer LNCaP cells, apigenin resulted in G1 arrest of cell cycle progression. Apigenin treatment markedly decreased the protein expression of cyclin D1, D2 and E and their activating partners CDK2, 4 and 6, and increased the expression of p21^WAF1/CIP1^ and p27^KIP1^ concomitantly. The induction of p21^WAF1/CIP1^ appears to be transcriptionally upregulated and p53 dependent [15]. And in an oral squamous cell carcinoma cell line SCC-25, apigenin treatment caused cell cycle arrest at both G0/G1 and G2/M checkpoints, associated with decreased expression of cyclin D1 and E, and inactivation of CDK1 [33]. Interestingly, Solmaz et al. reported that apigenin exposure induced G2/M arrest in imatinib-sensitive K562 cells while arresting imatinib-resistant K562/IMA3 cells in S phase especially at 100 μM apigenin [34]. Taken together, those data suggest that apigenin possibly modulates cell cycle progression in a dose-dependent and/or cell line specific manner.

### Induction of autophagy

Autophagy, the so-called type 2 non-apoptotic cell death, is characterized by the sequestration of cytoplasmic material into vacuoles for bulk degradation by lysosomal enzymes. Autophagy is a dynamic process where the cell digests its own cytoplasmic materials within lysosomes and results in the sequestration and degradation of macromolecules [35, 36]. There is growing evidence that the relationship between autophagy and cancer is complex and contradictory. In some cases, autophagy can serve as a cell survival pathway by providing recycled metabolic substrates and maintaining energy homeostasis during starvation, while in other settings, it can cause cell death, either in collaboration with apoptosis or as a backup mechanism.

Autophagy triggered by apigenin was first observed in erythroleukemia TF1 cells. Apigenin treatment triggered the initiation of autophagy without apoptosis [37]. Since then, more evidences have been presented that apigenin could induce autophagy which serves as tumor suppressive or tumor protective role under different circumstances [38, 39].

Tong et al. reported that apigenin exerted its chemopreventive by inducing autophagy in human keratinocytes via activation of AMPK [40]. In human breast cancer T47D and MDA-MB-231 cells, Cao et al. found that apigenin exposure triggered cell apoptosis and autophagy as evidenced by the accumulation of acidic vesicular organelles (AVOs) and LC3-II, a marker of Atg5/Atg7 dependent autophagy. Further, the authors found that treatment with autophagy inhibitor of 3-MA significantly enhanced apigenin-triggered apoptosis, suggesting that autophagy induced by apigenin play a tumor protective role in apigenin-caused cytotoxicity [41]. Similarly, in human colon cancer HCT116 cells, Lee et al. proved that apigenin concomitantly caused apoptosis and autophagy. And autophagy played a cell protective role in apigenin-induced cell apoptosis as well [28].

Beclin-1 regulates the dynamic autophagic process via the formation of autophagosomes [42, 43]. Beclin-1 is frequently downregulated in many types of cancers, including solid Ehrlich carcinoma. Gaballah et al. found that combining 5-FU with apigenin significantly increased Beclin-1 compared with the vehicle-treated control mice [44]. In addition, Wang et al. showed that apigenin treatment induced autophagy in macrophages as evidenced by upregulation of Beclin 1, Atg5, Atg7 and the appearance of LC3-II. And autophagy inhibition by 3-MA pretreatment significantly increased apigenin-induced apoptosis, further demonstrating that the autophagy triggered by apigenin protected macrophages from apigenin-induced cytotoxicity [45].

In contrast, in human papillary thyroid carcinoma BCPAP cells, apigenin exposure resulted in autophagic cell death associated with p62 degradation and Beclin-1 accumulation and LC3 protein conversion. Interestingly, co-treatment with 3-MA significantly protected apigenin-induced cytotoxicity, indicating that apigenin-induced autophagy here is more likely to be a tumor suppressor [32].

Together, the role of autophagy in apigenin-induced cytotoxicity depends on cancer cell types. In most reports, the apigenin-triggered autophagy functions to mediate the acquired resistance of cancer cells against cell apoptosis, evidenced as enhanced cell apoptosis induced by apigenin when in cotreatment with autophagy inhibitors. Under this circumstance, the autophagy plays cytoprotective roles in apigenin-induced cytotoxicity in cancer cells. In contrast, autophagy acts as an executioner by inducing autophagic cell death in human papillary thyroid carcinoma BCPAP cells [32].

### Inhibition of cancer cell migration and invasion

Based on growth site, tissue origin and growth characteristics, tumors can be divided into benign tumors and malignant tumors. Benign tumor cells do not have the ability to migrate. These cells grow and form lesions only in the primary site of the tumor and can be removed through clinical surgery. However, malignant tumor cells are highly unstable and have the ability to metastasize and invade other tissue to form further lesions. The majority of patients in clinical practice do not die of primary disease but by varying degrees of tumor metastasis. Presently, metastases, along with the development of chemoresistance and tumor relapse, are still the major barriers to effective treatment with cancer therapy. Apigenin has been shown to inhibit cancer cell migration and invasion in in vitro cancer cells and in vivo animal models.

In prostate cancer DU145 cells, apigenin strongly inhibited tumor cell invasion and migration in a dose-dependent manner [46]. With human malignant melanoma cells, 40 µM apigenin significantly inhibited cell migration and invasion though the AKT/mTOR pathway in melanoma A375 and C8161 cell lines [14]. In the human lung cancer cell line A549, apigenin exerted anti-migration and anti-invasion effects by suppressing the phosphorylation of AKT and targeting the PI3K/AKT signaling pathway [47]. And in the colorectal cancer cell lines DLD1 and SW480, Dai et al. proved that apigenin could inhibit cell migration, invasion, and metastasis through modulating the NEDD9/Src/AKT cascade [48].

Moreover, in human ovarian cancer A2780 cells, apigenin inhibited cancer cell migration and invasion by decreasing FAK expression in vitro and inhibited spontaneous metastasis of A2780 cells implanted into the ovary of nude mice in vivo [49]. In addition, in an orthotopic colorectal cancer model, apigenin prevented cell proliferation and migration by upregulating transgelin and downregulating MMP-9 expression by decreasing the phosphorylation of AKT; thus, apigenin inhibited tumor growth and metastasis to the liver and lung [50].

### Induction of immune responses

Cancer immunotherapy is a means of treating cancers by activating the patient’s own immune system. Avoiding destruction by the immune system is a crucial characteristic for carcinoma cells in overcoming human immune system surveillance [51]. The programmed cell death 1 (PD1) protein is commonly expressed in immune cells, such as T cells, B cells, monocytes and natural killer cells [52, 53]. Its receptors, programmed death ligand 1 (PD-L1) and 2 (PD-L2), are commonly expressed on the surface of dendritic cells or macrophages [54, 55]. Recognition and interaction between PD1 and its ligand will activate PD1 signaling in T cells and blunt the T cell immune response. Therefore, the PD1/PD-L1 system ensures that the immune system is activated only at the appropriate time and place and minimizes the possibility of autoimmune inflammation [56]. PD-L1 is also observed to be highly expressed in many types of cancer cells and to contribute to cancer cell immune evasion [57]. Therefore, one of the strategies to stimulate immune surveillance against cancer cells is to target the expression of PD1/PD-L1 in cancer cells. In human and mouse mammary carcinoma cells, Coombs et al. proved that apigenin could target STAT1, resulting in the inhibition of IFN-γ-induced PD-L1 expression. Meanwhile, apigenin treatment induced PD1-expressing Jurkat T cell proliferation and interleukin-2 synthesis when co-cultured with MDA-MB-468 cells [58].

Another strategy for cancer cells to evade immune destruction is to inhibit effector T cells by favoring the development of T-regulatory cells (Tregs) [59]. In a murine pancreatic cancer model, apigenin treatment enhanced CD4+CD8+ T cells and decreased the percentage of Tregs, improving mouse survival time, reducing tumor weights and preventing splenomegaly. Studies have shown that apigenin potentially stabilized Ikaros expression in vitro and in vivo by targeting CK2 [60].

Furthermore, apigenin feeding for 2 weeks resulted in significant suppression of total immunoglobulin (Ig) E levels, whereas the levels of IgG, IgM and IgA were not affected in C57BL/6 mice. In addition, apigenin feeding further resulted in the decreased production of regulated-on-activation normal T cell expressed and secreted (RANTES) and the soluble tumor necrosis factor receptor I in mouse serum [61]. In addition, studies have shown that TC-1 tumor-bearing mice that were treated with apigenin combined with E7-HSP70 DNA were found to generate significant effector and memory E7-specific CD8+ T cell immune responses, thus generating strong therapeutic anti-tumor effects [62]. Taken together, these findings demonstrate that apigenin plays a role in cancer immunotherapy. Apigenin shows promise as a cancer immunotherapy agent by modulating PD1/PD-L1 expression in cancer/T killer cells and by regulating the percentage of T killer and T regulatory cells.

### Apigenin functions on cancer stem cells

Apigenin shows significant cell cytotoxicity selectively against various types of cancer cells with low or no toxicity to normal cells. These selective anti-cancer effects are further shown to suppress cancer stem cells (CSCs) in various types of cancers. CSCs are closely associated with drug resistance, metastasis and the recurrence of cancer. In the head and neck squamous cell carcinoma cell lines HN-8, HN-30 and HSC-3, apigenin significantly downregulated the stem cell markers of CD44, NANOG, and CD105, and abolished the hypoxia-induced increase in CD44(+) cells, CD105(+) cells and STRO-1(+) cells [63]. In human glioblastoma cells, apigenin inhibited both the self-renewal capacity, such as cell growth and clonogenicity, and the invasiveness of GBM stem-like cells by blocking the activation of c-Met signaling [64]. In addition, in CD44(+) prostate CSCs of PC3 cells, apigenin dose-dependently inhibited cell survival and proliferation by inducing extrinsic cell apoptosis and increasing cell cycle arrest. Apigenin also suppressed stem cell migration and adhesion by downregulating matrix metallopeptidases-2, -9, Snail and Slug. Meanwhile, apigenin treatment significantly downregulated stemness marker Oct3/4 protein expression by downregulation of PI3K/Akt and NF-κB signaling pathways. [65]. Sphere-forming cells (SFCs) have self-renewal capacity and possess stem-like cell properties. Apigenin was demonstrated to downregulate CK2α expression and inhibited the self-renewal capacity of SFCs in SKOV3 and HeLa cells [66, 67]. Meanwhile, by targeting CK2, apigenin synergistically enhanced PI3K/AKT inhibitor-induced apoptosis in CD34(+)CD38(−) leukemia cells without harming healthy hematopoietic stem cells [68]. Apigenin shows clear anti-cancer effects by inhibiting the self-renewal capacity of CSCs. This evidence further demonstrated the effective anti-cancer activities of apigenin. We have noticed that the current studies of the effects of apigenin on CSCs are mainly phenomenon descriptions rather than mechanism analyses. However, further studies of apigenin in cancer therapy are warranted.

## Signaling transduction modulation by apigenin in cancer therapy

Tumorigenesis is tightly correlated with gene mutation and aberrant cell signaling transduction. Mutated genes, such as EGFR and Kras, serve as oncogenes, resulting in the activation of their downstream signaling components and driving the malignant transformation of normal cells. Therefore, oncogenes and their downstream signaling pathways are effective targets for cancer therapy. Apigenin has been reported to target multiple signaling pathways and has been shown as a promising chemotherapy agent against cancer.

### PI3K/AKT/mTOR signaling pathway

The phosphatidylinositol 3-kinase (PI3K)/AKT/mammalian target of rapamycin (mTOR) pathway is one of the most commonly activated signaling pathways, playing a central role in cell growth, proliferation, migration and differentiation [69–71]. Aberrant activation of these pathways has been linked to cancer development and is frequently detected in malignancies. Once activated, AKT phosphorylates a broad range of proteins involved in apoptosis, cell cycle regulation, growth and survival [72]. Apigenin has been shown to inhibit AKT function in different cancer cell types by directly suppressing PI3K activity by blocking the ATP-binding site of PI3K and subsequently inhibiting AKT kinase activity [73].

Moreover, Ultraviolet B (UVB) radiation is the major carcinogen for non-melanoma skin cancer by activating PI3K/AKT/mTOR signaling. Bridgeman et al. demonstrated that apigenin exposure significantly inhibited UVB-induced mTOR activation and enhanced UVB-induced autophagy to decrease cell proliferation in mouse keratinocytes. Interestingly, the mTOR inhibition by apigenin is driven by AMPK activation but not AKT-dependent [74].

Forkhead box O3 (FOXO3a), a transcription factor and tumor suppressor, is one of downstream targets of the PI3K/AKT signaling pathways and is negatively regulated by AKT. Activation of PI3K/AKT causes FOXO3a phosphorylation, which is related to poor prognosis in a broad spectrum of cancers [75]. In human breast cancer cells, falvone of apigenin and luteolin treatment induced FOXO3a expression by suppressing AKT phosphorylation, and subsequently upregulated the expression of FOXO3a target genes of p21^WAF1/CIP1^ and p27^KIP1^, which resulted in the inhibition of breast cancer cell proliferation [76]. In addition, apigenin inhibited the human PI3K/AKT/FOXO signaling pathway in human prostate cancer resulting in cell cycle arrest and cell apoptosis [77].

Hypoxia is a shortcoming of radiotherapy in malignant cancers, including laryngeal carcinoma. GLUT-1 is an important marker in hypoxia-induced therapies. Apigenin has the potential to decrease GLUT-1 expression levels via downregulation of the PI3K/AKT signaling pathway in vitro and in vivo, which enhances xenograft radiosensitivity and inhibits tumor growth [78].

Interestingly, apigenin can activate PI3K/AKT/mTOR signaling pathway and protect cardiomyocytes from chemotherapy-caused cytotoxicity in mice. Adriamycin is widely used in clinic for treatment of various types of cancers. However, the severe cardiotoxic side effects caused by adriamycin limited its usage in cancer therapy. Yu et al. reported that apigenin alleviated adriamycin-induced cardiotoxic by activating PI3K/AKT/mTOR pathway which inhibited adriamycin-induced cardiomyocyte apoptosis and autophagy [79]. Those data further demonstrate that apigenin has selective anti-cancer efficacy and low or no side effects to normal cells.

### MAPK/ERK signaling

The mitogen-activated protein kinase (MAPK)/extracellular signal-regulated kinase (ERK) signaling pathway is another oncogenic pathway that is frequently hyperactivated in cancer, deregulating the control of metabolism, cell apoptosis, survival and proliferation [80]. The mutation or overexpression of receptor tyrosine kinases and Ras will inevitably cause hyperactivation of this pathway. Therefore, the components of this signaling pathway are ideal therapeutic targets for cancer therapy. In addition to inhibiting the PI3K/AKT signaling pathway, apigenin was proven to modulate MAPK/ERK signaling pathway in various cancers in vitro and in vivo. In human melanoma A375 and C8161 cell lines, apigenin effectively suppressed cell proliferation, migration and invasion and induced G2/M phase arrest and apoptosis via decreasing p-ERK1/2, p-AKT and p-mTOR [14]. In non-small cell lung cancer cells, apigenin enhanced TRAIL-induced apoptosis by modulating DR4/DR5, AKT, ERK and NF-κB signaling [26]. And in the colorectal cancer cell lines HCT116 and DLD1, apigenin was found to enhance ABT-263-induced antitumor activity via the inhibition of the prosurvival regulators AKT and ERK in vitro and in vivo [24]. In addition, the co-inhibition of AKT and ERK signaling was observed in an autochthonous mouse prostate cancer model. Apigenin administration effectively suppressed prostate cancer progression in those mice by decreasing IGF/IGFBP-3 and inhibiting p-AKT and p-ERK1/2 [81].

In other studies, ERK signaling pathway was inhibited by apigenin along with other protein kinases, such as focal adhesion kinase (FAK). Hasnat et al. showed that apigenin induced anoikis in human cutaneous melanoma cells by reducing integrin protein levels and inhibiting the phosphorylation of both FAK and ERK1/2 [82]. In addition, in pancreatic cancer cells, apigenin targeted FAK and ERK to suppress the effects of 4-(methylnitrosamino)-1-(3-pyridyl)-1-butanone on pancreatic cancer cell proliferation and migration [83].

Meanwhile, ERK was not inhibited but activated by apigenin in few studies. In human prostate cancer LNCaP and PC-3 cells, Shukla et al. demonstrated that apigenin treatment increased phosphorylation of ERK1/2 and JNK1/2 and decreased phosphorylation of p38. The modulation of MAPKs by apigenin contributed to apigenin-induced cell cycle arrest at G0/G1 phase [84]. And in human choriocarcinoma cells, apigenin was found to induce cell apoptosis and reduce cell survival by suppressing the AKT-mTOR pathway and increasing the phosphorylation of ERK1/2 and P90RSK in a dose-dependent manner [85].

### NF-κB signaling pathway

The Nuclear Factor-κappa B (NF-κB) signaling pathway is generally considered an active factor in survival and proliferation. There are several homodimer and heterodimer forms of NF-κB: NF-κB1 (p50/p105), NF-κB2 (p52/100), RelA (p65), RelB, and the c-Rel proteins [86]. In addition, the NF-κB family members are regulated by the IκB protein members (IκBα, IκBβ, IκBε, IkBγ, Bcl-3, p100, and p105). NF-κB is a heterodimer in the cytoplasm and binds to IκB in an inactive state. Many different signal molecules, such as TNF, FasL and TRAIL, cause IKK complex activation, resulting in IκBα phosphorylation and degradation by the proteasome. Then, NF-κB is released and translocated to the nucleus. NF-κB inhibits cell death via the activation of target genes, which include prosurvival genes (Bcl-2, Bcl-xL, survivin, XIAP), cell cycle-related genes (cyclin D1), VEGF, inflammatory cytokines, and tumor metastasis genes (COX-2) [87, 88].

In most cases, apigenin treatment inhibits NF-κB activation both in vitro and in vivo. In a prostate TRAMP mouse model, Shukla et al. showed that apigenin feeding to TRAMP mice inhibited prostate tumorigenesis by interfering with NF-κB signaling. Apigenin administration significantly decreased prostate tumor volumes and completely abolished cancer cell metastasis. Studies have shown that apigenin administration blocked the phosphorylation and degradation of IκBα by inhibiting IKK activation, which in turn led to the suppression of NF-κB activation [89]. Further, Shukla and colleagues proved that apigenin was a specific IKK inhibitor by directly binding with IKKα to attenuate its kinase activity, thereby suppressing NF-κB/p65 activation in the human prostate cancer cell lines PC-3 and 22Rv1. In addition, the inhibitory efficacy of apigenin on IKKα is much more effective than PS1145, a specific IKK inhibitor [90].

Moreover, in the human non-small cell lung cancer cell line A549, apigenin did not affect the expression of NF-κB but suppressed the translocation of NF-κB from the cytoplasm to the nucleus, which further inhibited target genes, such as Bcl-2, Mcl-1 and Bcl-xL, that block apoptosis. Apigenin also blocks the degradation of IκBα in lung cancers, which further blocks the separation of IκBα from the NF-κB heterodimer [26]. And in malignant mesothelioma, apigenin treatment showed anti-cancer effects in vitro and in vivo by inhibiting NF-κB nuclear translocation and AKT activation and modulating MAPK signaling pathways [91].

### JAK/STAT signaling

The signal transducer and activator of transcription (STAT) proteins are members of a family of transcription factors that mediate signals from cytokines and growth factors to regulate cell proliferation and differentiation. STAT activation is usually mediated by non-receptor tyrosine kinase members of the Janus kinase (JAK) family [92]. JAK/STAT signaling is constantly activated in various human carcinomas and promotes tumorigenesis and metastasis by promoting the expression of genes that encode antiapoptotic proteins, cell cycle regulators, and angiogenic factors [93, 94]. Moreover, STAT3 can also be activated by tyrosine kinase receptors, such as EGFR and c-MET [95, 96]. Therefore, targeting STAT members is considered and evaluated as a promising therapeutic strategy in various cancer therapies.

In the murine melanoma B16F10 cell line, apigenin showed anti-metastatic activity via STAT3 phosphorylation inhibition. Meanwhile, the authors found that apigenin downregulated the STAT3 target genes MMP-2, MMP-9, VEGF and Twist1, which are important for cell migration and invasion [97]. In human myeloid leukemia HL60 cells and erythroid leukemia TF1 cells, the JAK/STAT pathway was one of the major targets of apigenin. Apigenin decreased the phosphorylation of JAK2 and STAT3 in HL60 and TF1 cells and decreased STAT5 in TF1 cells. The decrease in JAK/STAT phosphorylation enhanced apigenin-induced leukemia cytotoxicity [37]. Additionally, in the human HER2-overexpressing breast cancer cell lines BT-474, SKBR3 and MDA-MB-453, Seo et al. found that apigenin triggered cell apoptosis by suppressing JAK/STAT3 signaling and decreasing nuclear translocation of STAT3 [98, 99]. Therefore, apigenin is one of the agents that can effectively target STAT signaling pathways.

Besides, JAK/STAT signaling pathway was not affected by apigenin in some reports. In human ovarian cancer SKOV3 cells and the chemoresistant ovarian cancer SKOV3/TR cells, apigenin significantly decreased Axl and Tyro3 receptor tyrosine kinase at both RNA and protein levels, without changing the IL-6 production and phospho-STAT3 protein levels [100].

### Wnt/β-catenin signaling

Wnt/β-catenin signaling is highly conserved from sponge to human and plays important roles in metazoan development and tissue homeostasis. Dysregulation of this signaling pathway leads to the accumulation of β-catenin in the nucleus and is linked to several human diseases, including cancer [101, 102]. The increased expression of Wnt, frizzle or lymphoid enhancer factor (LEF)/T cell factor (TCF) in this signaling pathway is commonly detected in patients with leukemia, colorectal cancer, breast cancer or adrenocortical tumors [103–106]. In addition, targeting Wnt/β-catenin signaling by shRNA or the overexpression of domain-negative β-catenin or TCF has been found to suppress tumor cell growth and has become a new strategy for cancer treatment [107]. Apigenin was found to significantly inhibit the Wnt/β-catenin signaling pathway, thereby suppressing cell proliferation, migration, and invasion in colorectal and osteosarcoma cancers [11, 108]. Recently, Lin et al. reported that apigenin downregulated total, cytoplasmic and nuclear β-catenin through the induction of the autophagy-lysosomal system. Furthermore, they proved that the autolysosomal degradation of β-catenin by apigenin occurred via inhibition of the AKT/mTOR signaling pathway. In addition, treatment with the autophagy inhibitors wortmannin and chloroquine restored the accumulation of β-catenin in the cell nucleus, indicating the involvement of the autophagy-lysosomal system in the degradation of β-catenin [109].

In addition to the signaling pathways mentioned above, there is evidence of apigenin involvement in other signaling pathways. Those signaling pathways include AMPK [40], transforming growth factor-β (TGF-β) [110, 111], JNK [44, 112] and FAK [82, 83]. All of these proteins and signaling pathways are potential therapeutic targets for cancer treatment. Apigenin functions as a promising chemotherapy agent that is able to effectively target multiple signaling pathways. And the modulation of these signaling pathways by apigenin induces cancer cell apoptosis or autophagy and attenuates cancer cell proliferation or metastasis.

## Combination therapy for apigenin

Given that cancer cells have multiple genetic alterations, a combinatorial therapeutic strategy is demanded for effective cancer therapy. The main purposes of the combinatorial strategy for cancer therapy are to potentiate the antitumor effects of chemotherapeutic agents and to overcome the limitation of acquired drug resistance. Apigenin is an effective anti-cancer agent but with only moderate anti-cancer efficacy when used alone at human physiological dosages [24, 38, 113]. Therefore, co-treatment with other chemodrugs is a reasonable way to enhance its anti-cancer activities. The combination of apigenin and other chemodrugs are summarized in Table 2. In addition, most of the combination treatments resulted in enhanced anti-cancer efficacy in vitro and in vivo.

**Table 2:** The combination therapy by apigenin and other chemodrugs

Cotreatment partner	Tumor type	Cell lines (concentration)	Combination effects	Mechanisms	Citations
IFNγ	Cervical cancer	HeLa and SiHa (10 μM)	Enhance the anticancer activity	Targeting cyclin-dependent kinase 1	[114]
Paclitaxel	Ovarian cancer	SKOV3 (40 μM)	Overcome taxol resistance	Downregulation of Axl and Tyro3 RTKs expression	[100]
Cisplatin	Multiple tumor types	HeLa, A549, HCT 116, H1299, and MCF-7 (30 µM)	Enhances the cisplatin cytotoxic effect	Increased DNA damage in a p53-dependent manner	[116]
	Prostate cancer stem cells	PC3 and CSCs (15 μM)	Enhance anticancer effects	Suppressed PI3K/AKT activation and protein expression of NF-κB	[117]
	Laryngeal carcinoma	Hep-2 (40 μM)	Enhance the sensitivity to cisplatin	Inhibition of GLUT-1 and p-AKT	[119]
	Solid Ehrlich carcinoma	Swiss male albino mice, intraperitoneally (100 mg/kg)	Enhanced anti-cancer effect	Increased Beclin-1, caspases 3, 9 and JNK activities and decreased Mcl-1	[44]
5-Fluorouracil (5-FU)	Hepatocellular carcinoma	SK-Hep-1 and BEL-7402 (4 μM)	Enhanced anticancer activity	Inhibition of ROS-mediated drug resistance and decreased Bcl-2 expression and loss of ΔΨm	[120]
	Pancreatic cancer	BxPC-3 (13 μM)	Potentiate anti-proliferative effect	Decreased nuclear GSK-3β and NF-κB p65	[121]
Doxorubicin and etoposide	Leukaemia	CCRF-CEM and Jurkat (10 μM)	Enhancing cell cytotoxicity	Increased DNA damage	[122]
TRAIL	Non-small cell lung cancer	A549 and H1299 (20 μM)	Enhance anti-tumor activity	Upregulated DR4/DR5 expression in a p53-dependent manner	[26]
	Anaplastic thyroid carcinoma	8505C and CAL62 (40 μM)	Potentiates synergistic cytotoxicity	Reduced Bcl-2 and inactivation of ERK	[125]
	Prostate cancer	DU145 (20 μM)	Enhancing cell apoptosis	Targeting adenine nucleotide translocase-2	[124]
ABT-263	Colon cancer	HCT116 and DLD1 (20 µM)	Enhance cell apoptosis	Inhibition of AKT and ERK signaling and Mcl-1 and upregulation of Bim	[24]
miR-433-5p knockdown	Glioma stem cell	CD133-positive GSCs (20 μM)	Enhance cell apoptosis	Changes in Bax/Bcl-2 ratio, increased cytochrome c level, Apaf-1 induction, and caspase-3 activation	[126]
miR-138	Neuroblastoma	SK-N-DZ and SK-N-BE2 (100 μM)	Enhance cell apoptosis inhibition of cell viability	Increased Bax/Bcl-2 ratio and caspase-3,8	[127]
4-Hydroxy-2-nonenal (4-HNE)	The rat adrenal pheochromocytoma	PC12 (20 μM)	Attenuate 4-HNE-mediated cell death	Restore 4-HNE-induced ER homeostasis through modulating of UPR, Nrf2-ARE and MAPK pathways	[128]

Chemotherapy drugs, such as cisplatin and paclitaxel, are widely used in the clinic for cancer control. These drugs play a considerable role in the extension of the overall survival rates of cancer patients; however, their undesired toxicity has always been a matter of concern for clinicians and patients. To enhance their antitumor effects and to minimize their limitation, co-administration with other targeted drugs has been widely tested and has achieved great success in clinical applications. Studies have shown that co-administration with apigenin significantly enhances the anti-cancer efficacy of chemodrugs and helps overcome their limitations in various types of cancers by targeting multiple signaling pathways (Table 2) [44, 114–122].

Recombinant Apo2L/tumor necrosis factor-related apoptosis-inducing ligand (TRAIL) is an effective antitumor agent that induces cancer cell death without damaging normal cells and that has been evaluated in clinical trials. However, TRAIL treatment only showed limited anti-cancer activity in many malignant cancers because of acquired resistance [123]. Overcoming this resistance is essential for chemotherapy using the Apo2L/TRAIL pathway. In prostate cancer DU145 and LNCaP cells, apigenin overcomes resistance to Apo2L/TRAIL by inhibiting adenine nucleotide translocase-2 (ANT2) and upregulating DR5. Further, silencing of ANT2 by siRNA lowered the enhancement of DR5 expression by apigenin, indicating that ANT2 inhibition is needed for apigenin to enhance DR5 expression and Apo2L/TRAIL-induced apoptosis [26, 124]. NSCLC A549 and H1299 cells are resistant to TRAIL treatment alone. Apigenin exposure upregulates DR4 and DR5 expression and sensitizes those cells to TRAIL-induced apoptosis in a p53-dependent manner [26, 124]. Furthermore, Kim et al. showed that apigenin synergistically enhanced the cytotoxicity of TRAIL in anaplastic thyroid carcinoma (ATC) cells by modulating the Bcl-2 family proteins [125].

MicroRNAs (miRNAs) are short non-coding RNAs of 20–24 nucleotides that function in post-transcriptional regulation of gene expression. Aberrant miRNA expression may affect a multitude of transcripts and profoundly influence cancer-related signaling pathways. Therefore, miRNAs may function as tumor suppressors or oncogenes involved in the pathogenesis of tumors. Modulation of miRNA expression could also enhance apigenin-induced antitumor effects. miR-423-5p is overexpressed in glioblastomas and contributes to glioma stem cells. The downregulation of miR-423-5p enhances apigenin-induced cell apoptosis in glioma stem cells with a shift in the Bax/Bcl-2 ratio, an increased cytochrome c level, Apaf-1 induction and caspase-3 activation [126]. In contrast, in malignant neuroblastoma SK-N-DZ and SK-N-BE2 cells, miR-138 overexpression significantly enhanced apigenin-induced cell apoptosis and decreased cell viability and colony formation capability in vitro and effectively suppressed tumor growth in vivo [127].

Alleviating the side effects of one drug by co-treatment with a second agent is also a widely used strategy in cancer therapy. It is known that apigenin exhibits a broad spectrum of biological activities, including antioxidant and anti-inflammatory activities. Nephrotoxicity is one of the adverse effects that limits the usage of cisplatin in cancer therapy. Hassan et al. found that co-administration with apigenin significantly reduced blood urea nitrogen, serum creatinine, TNF-α, IL-6, COXI, COXII, and MDA levels and increased GSH levels, thereby protecting Wistar Albino mice from cisplatin-induced nephrotoxicity [118]. One lipid peroxidation product that is implicated as a causative factor to cause neurodegenerative disorders is 4-hydroxy-2-nonenal (4-HNE). In another study, apigenin significantly attenuated 4-HNE-mediated cell death in neuronal-like catecholaminergic PC12 cells via restoration of ER homeostasis [128]. Therefore, apigenin can not only be used as an adjuvant chemotherapeutic agent to overcome drug resistant but also show significant protective effects and alleviate chemodrug-mediated adverse effects.

## Conclusions

As a naturally occurring flavonoid compound, apigenin not only has low toxicity characteristics but also plays an important role in a variety of ways. All evidence gathered thus far clearly indicates that apigenin has strong anti-cancer activities against various human cancers alone and in combination with other chemotherapeutic agents. It is worthy to note that in most cases apigenin treatment can concomitantly cause multiple anti-cancer effects in the same treatment. For example, In ACC cells, apigenin suppressed ACC-2 cell survival by inducing both apoptosis and G2/M-phase arrest in a dose- and time-dependent manner [28]. And in the human colon cancer HCT116 cells, apigenin treatment triggered both autophagy and apoptosis [28]. Furthermore, in human melanoma cells, Zhao et al. reported that apigenin showed effective antitumor effects as suppression of cell migration and invasion, induction of cell cycle arrest at G2/M phase and triggering cell apoptosis simultaneously [11]. These different antitumor effects simultaneously triggered by apigenin demonstrated that apigenin has a wide range of antitumor effects, but also the results that apigenin can simultaneously target a variety of signal pathways and protein kinase. As summarized in Fig. 2, the same signal pathway inhibition will also lead to different antitumor effects.

Apigenin shows antitumor activities by modulating multiple signaling pathways, including PI3K/AKT, NF-kB, JAK/STATs, Wnt/β-catenin, AMPK, MAPK/ERK, and JNK. We need to note that although so many signaling pathways are reported to be modulated by apigenin, it is still not clear whether there is cross regulation among those signaling pathways. In addition, it is unclear how apigenin functions to modulate those signaling pathways. To determine the direct targets of apigenin, Arango et al. carried out high-throughput screening of phage display coupled with second generation sequencing and identified a group of 160 potential targets of apigenin. Those targeted proteins are significantly enriched in three main functional categories: GTPase activation, membrane transport, and mRNA metabolism/alternative splicing [129]. However, whether the signaling pathways modulated by apigenin are regulated through those direct targets still needs further exploration.

Though apigenin was widely investigated with xenograft models in mice for its anticancer effects, few reports mentioned that apigenin caused side effects to animals. To better develop and utilize apigenin in cancer therapy, the potential toxicity of apigenin was investigated in Swiss mice with acute exposure test [130]. Apigenin was administered intraperitoneally at doses of 25, 50, 100 and 200 mg/kg. Twenty-four hours later, mice were sacrificed and blood and liver tissues were collected for further analysis. Singh et al. found that doses of 100 or 200 mg/kg but not 25 or 50 mg/kg apigenin showed liver toxicity, evidenced as increased ALT, AST, ALP in serum and increased ROS, ratio of oxidized to reduced glutathione (GSSG/GSH) and LPO, and altered enzyme activities along with damaged histoarchitecture in the liver tissue [130]. This warrants the doses of apigenin by intraperitoneal route in vivo.

Until now, the anticancer effects of apigenin have been mainly studied in in vitro cancer cells and preclinical animal models. There are still no clinical data on apigenin in human cancer therapy. The good news is that the pharmacological effects of apigenin as a dietary supplement are under evaluation in a phase 2 clinical study by Technische Universität Dresden. The bioflavonoid mixture, with 20 mg apigenin and 20 mg epigallocatechin gallate, is served as a daily nutritional supplement to patients with resected colorectal carcinomas to evaluate the prevention of the recurrence of neoplasia [https://clinicaltrials.gov/ct2/show/NCT00609310?term=Technische±Universit%C3%A4t±Dresden±apigenin&rank=1]. With more evidence, an in-depth understanding of apigenin in cancer therapy and more in-depth studies on its pharmacological mechanism and toxicology in different cancers, further clinical studies on apigenin in cancer therapy will be warranted. Apigenin appears to have the potential to be developed either as a dietary supplement or as an adjuvant chemotherapeutic agent for cancer therapy.

## Abbreviations

NSCLC: non-small cell lung cancer
DR4: death receptor 4
DR5: death receptor 5
CDK1: cyclin-telangiectasia mutated
GLUT-1: glucose transporter-1
ROS: reactive oxygen species
AVOs: acidic vesicular organelles
PD1: programmed cell death 1
PD-L1: programmed death ligand 1; P dependent kinase-1
PCNA: proliferating cell nuclear antigen
ACC: adenoid cystic carcinoma
ATM: ataxia
D-L2: programmed death ligand 2
Tregs: T-regulatory cells
Ig: immunoglobulin
RANTES: regulated-on-activation normal T cell expressed and secreted
PI3K: phosphatidylinositol 3-kinase
mTOR: mammalian target of rapamycin
UVB: ultraviolet B
FOXO3a: forkhead box O3
MAPK: mitogen-activated protein kinase
ERK: extracellular signal-regulated kinase
FAK: focal adhesion kinase
NF-κB: nuclear factor-κappa B
STAT: signal transducer and activator of transcription
JAK: Janus kinase
LEF: lymphoid enhancer factor
TCF: T cell factor
TGF-β: transforming growth factor-β
TRAIL: tumor necrosis factor-related apoptosis-inducing ligand
ANT2: adenine nucleotide translocase-2
ATC: anaplastic thyroid carcinoma
miRNAs: microRNAs
4-HNE: 4-hydroxy-2-nonenal
CSCs: cancer stem cells
SFCs: sphere-forming cells
